# Towards Enhancing the Potential of Injection Molding Tools through Optimized Close-Contour Cooling and Additive Manufacturing

**DOI:** 10.3390/ma14123434

**Published:** 2021-06-21

**Authors:** Sebastian Gries, Guillaume Meyer, Andreas Wonisch, Reinhard Jakobi, Christian Mittelstedt

**Affiliations:** 1 Institute for Lightweight Construction and Design, Department of Mechanical Engineering, Technical University Darmstadt, D-64287 Darmstadt, Germany; sebastian@gries.name (S.G.); christian.mittelstedt@klub.tu-darmstadt.de (C.M.); 2 BASF SE Simulation Engineering & Ultrasim®, Performance Materials Europe, BASF SE, E-PME/NN, D-67056 Ludwigshafen am Rhein, Germany; andreas.wonisch@basf.com (A.W.); reinhard.jakobi@basf.com (R.J.)

**Keywords:** additive manufacturing, powder bed fusion, optimization, injection molding, close-contour cooling

## Abstract

This work deals with the parametric optimization of the position and form of a conformal cooling used in the injection molding industry. Based on a literature survey, an optimization routine concerning the parameter optimization of cooling system designs was developed and implemented with the help of the software package Moldflow. The main objective of the optimization is to reduce the cooling time; the second is to obtain an optimized homogeneous temperature distribution over the complete tool surface. To enable a comparison of the new close-contour solution with a classical manufacturing process, an optimized cooling system simulation, based on a conventional manufacturing solution, was established. It can be shown that the optimized close-contour cooling design offers significant advantages that cannot be exploited using classical manufacturing. Finally, the additive manufacturing of a prototype in the framework of powder bed fusion is documented as a proof of concept.

## 1. Introduction

Injection molding is the most common fully automatized process for manufacturing thermoplastic parts with complex geometries [1]. Its economic efficiency is largely influenced by the thermal performance of the injection molding tool when the molding compound is injected into the tool cavity under high pressure and temperature. Accordingly, the tool cooling system directly determines the cycle time, which can account for up to 60% of the total processing time [2] depending on the complexity of the manufactured part and the compound. Naturally, the more rapid cooling of the part from processing temperature to demolding temperature reduces the overall cycle time, thus enabling higher production rates. However, the cooling system also has a direct influence on the resultant quality of the manufactured part: a non-optimal cooling system will lead to a larger variation in temperatures on the tool surface, resulting in non-uniform temperature fields and thus to non-uniform cooling rates of the part, resulting in anisotropic shrinkage and eventual warping, which is generally not qualitatively acceptable. Therefore, an investigation into optimizing the heat exchange between the molding compound and the tool cooling system is justified both to increase the quality of thermoplastic parts and to decrease production costs.

The aim of this paper is to document the development of a new close-contour cooling system for a metallic injection molding tool using dedicated structural optimization and the numerical modeling of the flow and heat transfer and the injection molding process, itself. Based on a literature survey, a parameter optimization of cooling system designs was performed with the help of the Moldflow software package (Autodesk Inc., Mill Valley, CA, USA, v2019, 2019). This optimization was used to establish a close-contour cooling system in a reference mold core. Finally, the manufacturing of a prototype is documented as a proof of concept before this paper closes with its summary and conclusions.

### 1.1. Cooling Systems of Injection Molding Tools for Thermoplastics

Conventional cooling systems in injection molding tools usually consist of straight drilled or milled holes connected to the cooling circuit, where the mold cores that are used for forming three-dimensional parts are especially challenging. In such cases, straight holes are drilled where the cooling medium (usually water or oil) reaches temperatures up to 300 °C [3,4]. The strongly varying distances between the cooling system and the mold surface lead naturally to non-uniform temperature patterns. This means that cooling systems that are manufactured using the classical subtractive method are generally not optimal for heat exchange or manufactured part quality.

**Figure 1:**
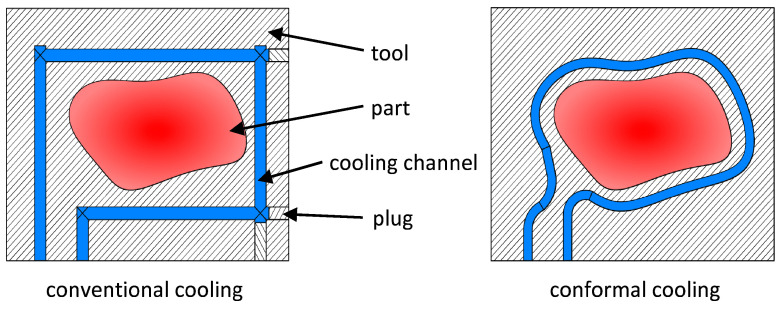
Schematic representation of a conventional cooling system (**left**) and a close-contour cooling system (**right**).

In a close-contour cooling system, the location and the pathways of the cooling channels follow the shape of the molded part closely, whereas in a conventional cooling system the channel locations are dictated by the available means of manufacturing. Figure 1 is a schematic representation of a conventional cooling system in comparison with a close-contour system. It has been shown that close-contour cooling can reduce cycle times by up to 40% [5].

**Figure 2:**
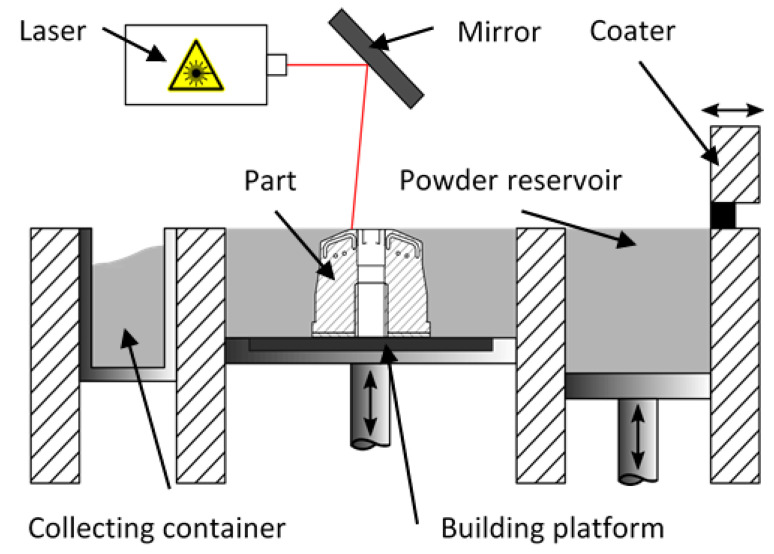
The Powder Bed Fusion (PBF) process.

Additive manufacturing, in the form of the so-called Powder Bed Fusion (PBF) process, is based on the layer-wise manufacturing of a structure by adding material rather than subtracting it as it is done in “classical” manufacturing [6,7,8] (Figure 2). Thus, PBF offers high flexibility and versatility with a theoretically unlimited degree of complexity that enables the manufacturing of structural parts out of any material that can be made into a powder. Consequently, PBF additive manufacturing has been established for the serial manufacturing of parts in many industries [7,9]. Regarding tooling machines, PBF enables the manufacturing of cooling systems that closely follow the contour of the injection molding tool, thus enabling an even temperature distribution.

Park and Dang [10] used a close-contour cooling system for steel injection molding tools to reduce the processing times of two molded automotive engineering parts in which additive manufacturing produced novel designs consisting of spiral and helix channels. A numerical simulation showed that processing times could be reduced by up to 31%, and an experimental validation amounted to 23% in time savings. Mazur et al. [11] employed PBF to manufacture a mold core with close-contour cooling and performed numerical and experimental comparisons with a core with conventional cooling. The experimental validation was performed by applying temperature sensors to the mold core. In all, the temperature distribution was more uniform and overall cooler than in the conventionally cooled mold core. Warping of the molded parts was also significantly reduced. Reis et al. [12] employed close-contour cooling for the tooling of a pipette adapter and found that cooling time could be reduced by about 49% where cooling in the initial design comprised about 93% of the entire processing time. The mold core was manufactured using PBF with steel CL 50WS. Brooks and Brigden [13] investigated the cooling capacity of additively manufactured cooling systems wherein the experimental setup consisted of an aluminum block as a heat source and a layer of cooling channels. It was found that the employed close-contour cooling system delivered significantly improved cooling rates compared to the conventional design.

It is worth mentioning that while all cited investigations proved the significant potential of close-contour cooling systems in injection molding tools for improved processing time and part quality, none performed a systematic mathematical programming optimization; rather, a heuristic design was manufactured and employed in numerical and experimental investigations. It is thus of importance to research the potential that can be reached when systematic optimizations are performed, which is the purpose of this paper.

### 1.2. Cooling System Optimization

The literature describes a good number of methods that had been successfully employed to optimize tool cooling, of which the majority of investigations were geared towards finding an optimal cooling system without a pre-existing design. The optimization approaches can be divided into deterministic methods, which take specific temperature distributions into account, and heuristic methods by which a close-contour cooling is developed based on simplified strategies without taking local temperature distributions into account.

Agazzi et al. [2] tackled an inverse heat transfer problem to establish optimized heat dissipation with uniform temperature distribution inside a molded part and its contour without having a preexisting cooling channel design. Conjugated gradients were used to optimize the fluid temperature using a twofold objective function. The first objective was to develop an efficient cooling system to shorten the time needed to reach a defined demolding temperature, while the second was to minimize the temperature gradients on the molded part’s surface for optimal quality. Hopmann and Nikoleizig [4] considered the inverse optimization problem of finding a homogeneous temperature and density distribution in the molded part and tool to minimize shrinkage and warping. The investigations necessitated an initial process simulation to determine the initial temperature in the tool and molded part. This served as the starting point for the optimizations where cooling system efficiency and machine-part quality comprised the objective function, Li et al. [15] employed the boundary element method to optimize the complex cooling channel network designs, which consisted of control points connected by linear cooling channels. For design variable, the optimization model used the locations of the control points and the channel diameters, which were restricted by upper and lower boundary values. The objective was to maximize the efficiency of the cooling system and establish a uniform temperature distribution.

Due to the high computational effort involved when using deterministic methods for generating temperature fields in the mold part and tooling, according to optimal close-contour cooling channel systems, a relatively large number of heuristic methods have been employed that rely on engineering intuition and systematic parametric studies to determine a channel distribution without resorting to mathematical programming [16]. In the simplest cases, such methods do not require numerical simulations and are thus very efficient. The main disadvantage is, of course, that although they usually lead to improved designs, they do not necessarily enable cooling systems that are optimal in every aspect. Wang et al. [17] employed Voronoi diagrams to decompose close-contour surfaces and used the edges of polyhedrons as positions for cooling channels. Afterwards, the channel distribution was smoothed by the use of a computer algorithm to improve the flow properties. Using Voronoi diagrams enabled a close and strongly interconnected mesh of cooling channels in the mold part contour but at the cost of local decreases in heat-transfer efficiency due to laminar flows. A later work by Wang et al. [18] documented the use of evenly distributed helix contours as cooling channel distributions, thus avoiding channel branches and the problems described in the preceding work. This methodology, however, is not applicable when intricate and complex mold parts and tool geometries are considered.

In summary, it can be stated that up to this day no optimization routine exists that can establish an optimized distribution of cooling channels without necessitating user interaction and interpretation of results. Furthermore, none of the discussed optimization routines accounted for the actual manufacturing process of the injection molding tool, which also holds for the additive PBF process. Consequently, any design that is the result of numerical simulation and optimization requires a certain amount of post-processing.

## 2. Methods

The optimization methodology used in this investigation is described briefly. For each calculation, the optimization software provides information about selected parameters for the creation and meshing of cooling channels in the pre-processing step. For the actual optimizations, the program LS-Opt (Livermore Software Technology Corporation, Livermore, CA, USA, v5.2, 2015) was employed, while the model pre-processing was performed using the open source CAD software FreeCAD (FreeCAD, www.freecadweb.org, v0.18, 2019), and ANSA (BETA CAE Systems International AG, Root Switzerland, v19.1.1, 2019), which has specific Python scripts to ensure compatibility among the different software systems. Then, the model was imported into the injection molding simulation software and the corresponding results are generated. The simulations were performed using the Autodesk Moldflow software package. Finally, the most relevant ones were extracted and sent back to the optimization software. The post-processing step was performed using Autodesk Moldflow and specific Python scripts for software compatibility purposes.

### 2.1. Reference Part and Initial Configuration

**Figure 3:**
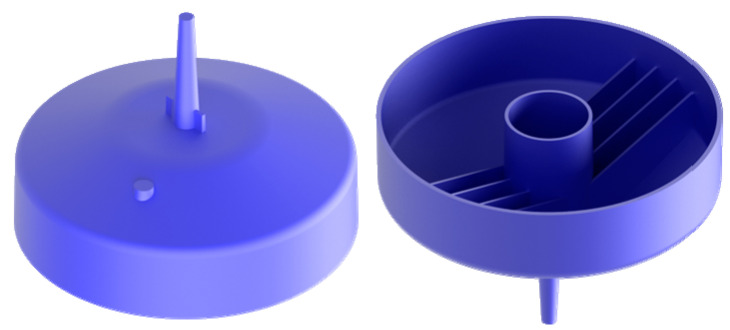
The reference molded part.

**Figure 4:**
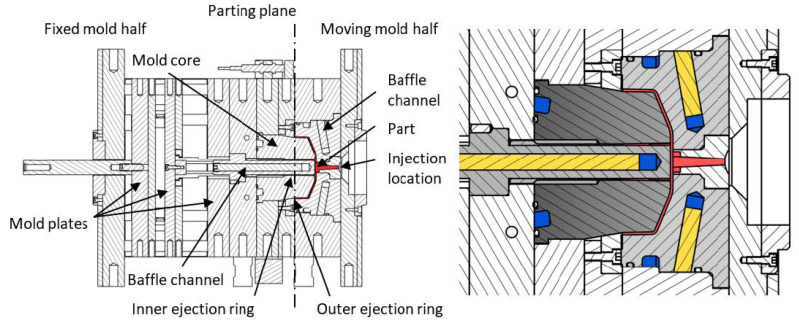
Experimental set-up: The injection molding tool (**left**); relevant detail of the tool for the cooling system (**right**). In the picture on the right, the molded part in the cavity is red; the mold core is dark grey; the inner core is light grey on the right; the die is light grey on the left. The observable cooling channels are in blue and divided by pass partition plates in yellow.

The reference part (Figure 3) was molded by BASF SE into a stiffened shell-like structure, a typical principle component for an experimental investigation into optimal cycle times for thermoplastics. The regions between the stiffening ribs are especially difficult for a cooling system to access, so an optimized integrated cooling system should be geared towards eliminating such so-called hot spots that naturally require a longer cooling time and thus determine the total cooling time. Figure 4 shows the experimental set-up including tooling, principle part and cooling channels.

**Figure 5:**
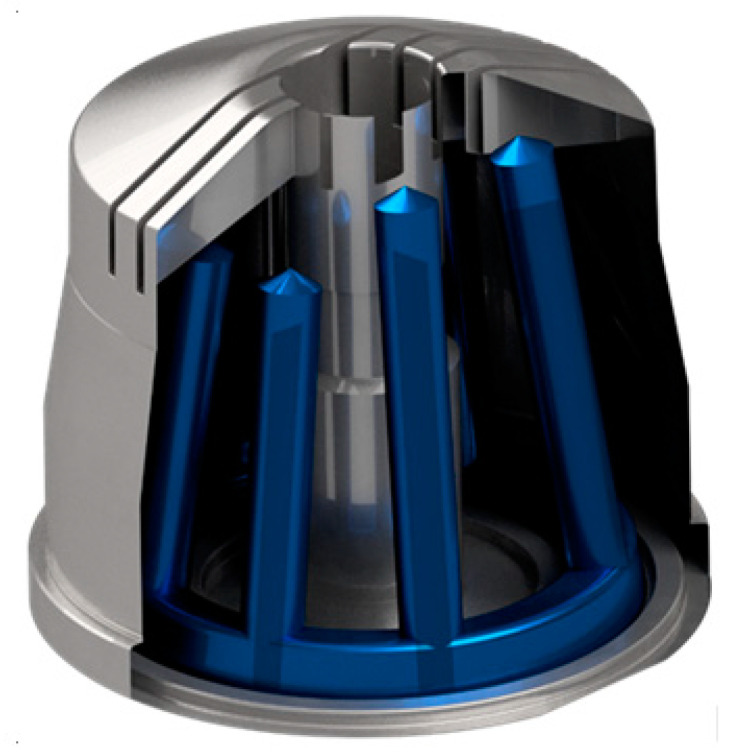
Sectional view of the solid mold core; cooling system consisting of channels with circular cross-sections, divided by pass partition plates. The cooling system consists of eight channels.

**Figure 6:**
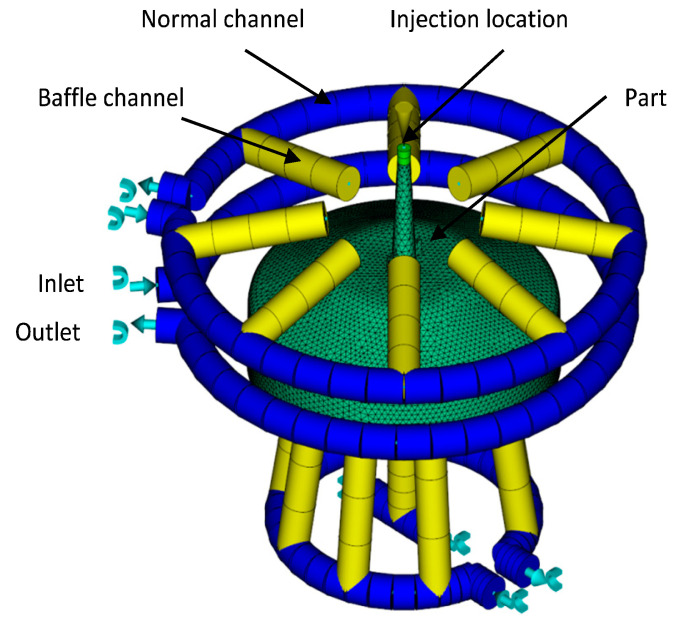
Model sketch of the initial configuration (mesh for tooling is omitted), cooling channels are given in blue; channels including pass partition plates are highlighted in yellow.

The current cooling system of the mold core is presented in Figure 5, and a model sketch for the given initial configuration of the injection molding tool with 4 cooling circuits is given in Figure 6. For the modeling approach, the center lines of the cooling channels are determined and discretized using one-dimensional line elements. The mesh for the mold part, which remained unchanged throughout the whole investigation, consisted of 577,933 tetrahedron elements. The properties of the material corresponded to standard mold construction steel 1.2311 according to the data available in the Moldflow material database. The relevant properties were thermal conductivity ë = 29 W/(mK); specific heat capacity c = 460 J/(kgK); and density *ρ* = 7800 kg/m^3^. The mold part was assumed to consist of the POM thermoplastic Ultraform^®^ N2320 003 (BASF SE, Ludwigshafen am Rhein, Germany). The process parameters were corresponded to test runs performed at BASF SE and are given in Table 1.

### 2.2. Non-Linear Optimization

Due to the high amount of calculation time involved in injection molding simulations, optimizations are performed on a meta model. To determine the investigation points of the meta model, a so-called space-filling algorithm was used. This method maximized the minimum distance between two arbitrary investigation points within the design space by means of heuristic optimization (adaptive simulated annealing) so that the maximum criterion was fulfilled [19,20,21]. The meta model worked on the radial basis function method [22]. The solution of the meta model was provided by the software package LS-OPT, which used the so-called Leapfrog optimizer for the constrained minimization (LFOPC) optimization algorithm [23]. This algorithm differs from other gradient methods since it does not look explicitly for lines but generates a dynamic trajectory from an arbitrary point that leads to a local optimum. The original Leapfrog method is based on the minimization of the potential energy of a particle in motion [24] and can lead to a global minimum by imposing penalties for a given starting point [21]. According to Stander and Craig [25], unless the abort criterion is reached, a new iteration is initiated through a reduction in the parameter space using the sequential response surface method (SRSM) whereby subfields of the problem area are considered separately and reduced for an optimal solution. Subsequently, the next iteration is performed on the newly generated subfield.

The aims and boundary conditions considered by subsequent optimizations are listed in Table 2. An optimized solution targets the maximization of the process efficiency by reducing the cycle time, while at the same time achieving a more uniform temperature distribution on the tool surface, which can be described by the standard deviation of the temperature inside the cavity. To reach a homogeneous temperature distribution, the difference between the temperatures at the cooling system inlet and outlet should be below 3 °C [14]. In the case of the present simulations, the end temperature was assumed to be reached when 99% of the volume of the mold part had a temperature at or below the required 110°C, which corresponds to the mold release temperature of the POM material. Furthermore, restrictions need to be considered concerning the mechanical loads of the mold core. Generally, injection molding tools are justified against quasi-static loadings [14], so to warrant the safety of the structure, and in the case of operating errors, the maximum possible injection pressure is used as a quasi-static load case [3]. In this paper the justification against mechanical loads is not considered further.

#### 2.2.1. Optimization of the Conventional Cooling System

**Figure 7:**
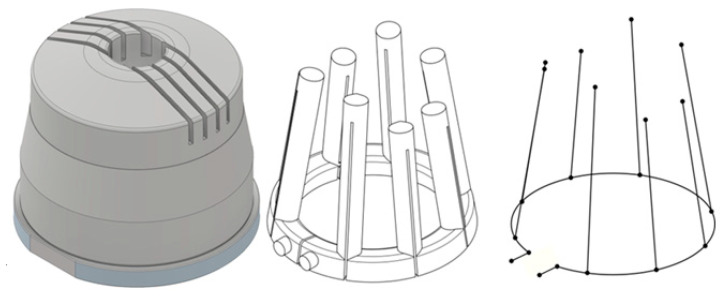
CAD model of the mold core (**left**), initial cooling system (**middle**), channel axes (**right**).

The optimization of a conventional cooling system was investigated for comparison purposes with a close-contour cooling. To do so, the optimization was performed for an arbitrary number of straight cooling channels. The CAD model of the existing mold core, the initial cooling system and the cooling channel axes, as required for boundary element method analysis, are given in Figure 7.

**Figure 8:**
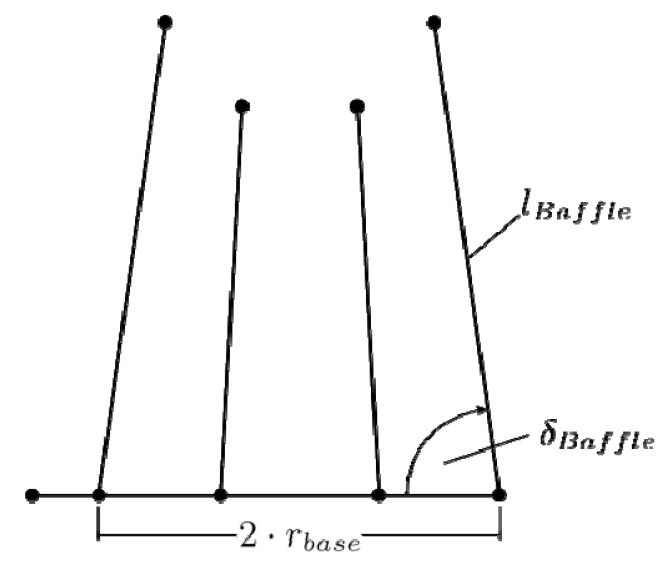
Graphical representation of the design variables for the optimization of the conventional cooling system.

The following optimizations are based on the design variables given in Table 3 and sketched in Figure 8. Specifically, we considered the length *l*_baffle_ of the pass partition plates, the number *n*_baffle_ of equidistant channels, the orientation angle *ä*_baffle_ of the channels, and the radius *r*_base_ of the base cooling channel. Restrictions for the design variables are outlined in Table 3 and mainly stem from constructive restraints imposed by the given situation. The process parameters given in Table 1 also hold for the current optimizations. The objective function in the current case is the cycle time required to attain a minimum.

#### 2.2.2. Optimization of a Close-Contour Cooling System

**Figure 9:**
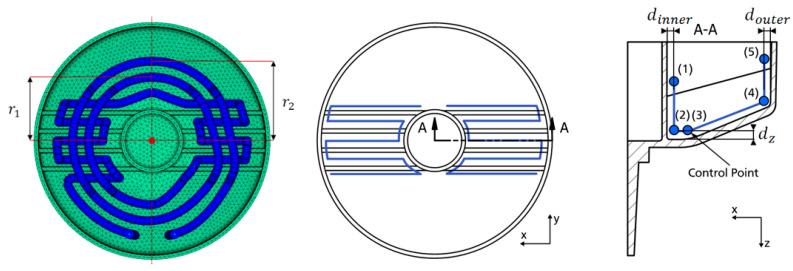
Initial close-contour cooling system in top view (**left**) and rib region parametrization (**middle** to **right**).

To assess the optimization potential of a close-contour cooling system, an initial parametrized design was established [26] (Figure 9) and gradually shifted towards an optimized solution. The parametrization drew from the conclusions of reference results and was steered by the geometry of the mold core. Furthermore, the symmetric geometry also enabled a significant a reduction in those parameters that described a close-contour cooling system. The distribution of the cooling channels was subdivided into 3 key regions: (a) the inlet and outlet, (b) the rib, and (c) the areal, which is outside the rib region. Positions of inlet and outlet channels were fixed and remained unchanged throughout all iterations. The cooling channel design was inspired by the zig-zag design of the close-contour cooling system as investigated by Park and Dang [10]. Figure 9, left, shows the lower surface of the mold part with the initial distribution of the cooling channels and control points. The parameters that required optimization are given in Table 3. The parameter design variable *d*_inner_ describes the distance of the two inner control points (1) and (2) from the inner hollow cylinder of the mold part, while *d*_outer_ is the distance of control from the outer hollow cylinder––points (4) and (5). Together with the distance *d*_z_, the cooling channel in the rib region is fully parametrized, and it is assumed that the cooling channels are always oriented parallel to the ribs. A minimum distance of 1.5 mm between the cooling channels and rib surfaces, corresponding to the remaining wall thickness, was taken into account at all times.

Using the preprocessor ANSA, curve generation was performed with B-splines with the help of intermediate control points (Figure 9, right). Contrary to the conventional design, the cooling system consisted of one continuous channel without the need for pass partition plates. From the inlet location, the cooling channel was oriented directly towards the rib region to supply it with the cooling medium at its coolest temperature. Afterwards, the cooling system assumed the shape of a helix with two radii: inner radius *r*_1_ and outer radius *r*_2_ (See Table 4). The current optimizations again used the process parameters as outlined in Table 1 with the exception of the flow rate, which required adjustment due to the potentially much smaller channel diameters of the close-contour cooling system. Here, a value of 1.6 L/min was used, which was the outcome from an unrelated internal BASF SE experiment on a close-contour steel tool with the original configuration from Wonisch et al. The material properties of the tool and mold part remain unchanged.

## 3. Results and Discussion

### 3.1. Initial Configuration

The simulation results are summarized in Table 5. The minimum cycle time for a reliable deformation of the mold part was 43.9 s, which included an injection time of 0.8 s, the holding-pressure time of 10 s and mold opening and closing of about 5 s. The remaining 28.1 s was the time that required for cooling the mold part so that it could be removed from the injection molding tool.

**Figure 10:**
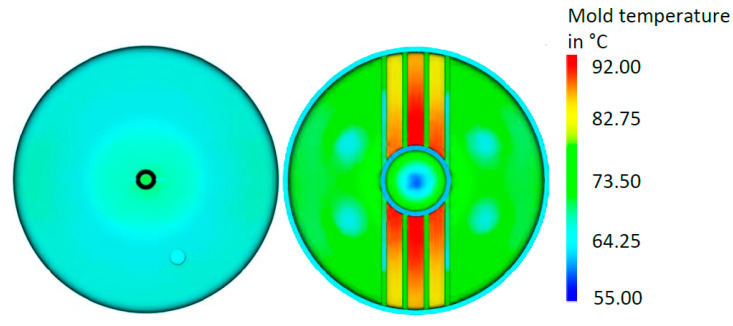
Averaged temperature distribution on the surfaces of the mold part for the initial configuration: Upper surface (**left**), lower surface (**right**).

The temperature distribution on the surfaces of the mold part, averaged over the cycle time, is shown in Figure 10. While a rather homogeneous temperature was found on the upper surface of the mold part (Figure 10, left), strong irregularities were found on the lower surface (Figure 10, right), especially in the vicinity of the ribs where significantly higher temperatures (around +60%) were encountered. This was attributed to the fact that the conventional cooling system could not reach the areas between the ribs, so the heat transfer there was much lower. The other regular areas showed a much more uniform temperature distribution; however, the temperatures were found to be distinctly higher than on the upper surface.

### 3.2. Optimized Conventional Cooling

**Figure 11:**
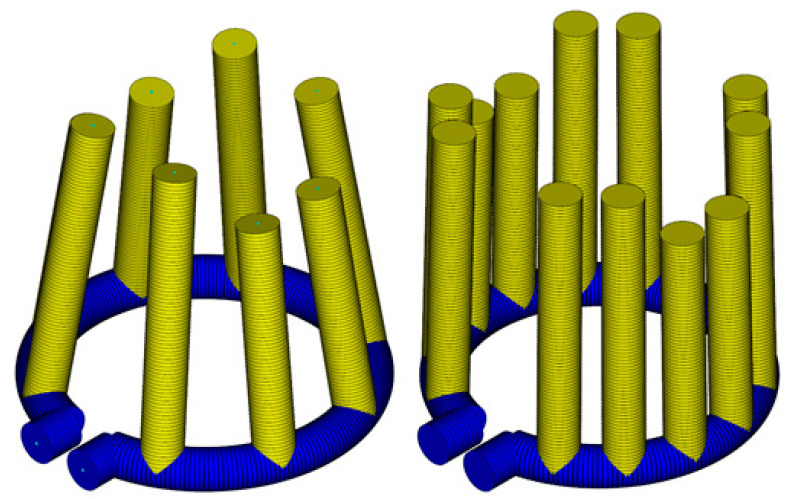
Initial configuration (**left**) and optimized configuration (**right**) of the conventional cooling system.

The optimal solution presented in the following section required 6 iterations with 84 simulations each. The initial configuration and the determined optimal solution are given in Figure 11. The number of cooling channels increased from 8 to 12 while the radius of the base channel decreased from 40.8 to 38.9 mm. The orientation angle increased from an initial value 81.0° to 88.8°, i.e., nearly vertical channels. The length of the channels at specific locations remains unchanged because the initial length also served as the upper boundary due to geometric factors. Apparently, a higher number of cooling channels translates into a higher cooling surface, leading to a quicker heat transfer from the cavity surfaces into the cooling medium.

The key results for the initial configuration and the optimized solution are summarized in Table 6. A 2.3% reduction in cycle time was reached, but it was not a significant improvement. The mean temperature in the cavity in the optimized solution was also slightly below the temperature of the initial configuration. Somewhat surprisingly, however, was that the maximum temperature increased by about 6%, which was not advantageous.

**Figure 12:**
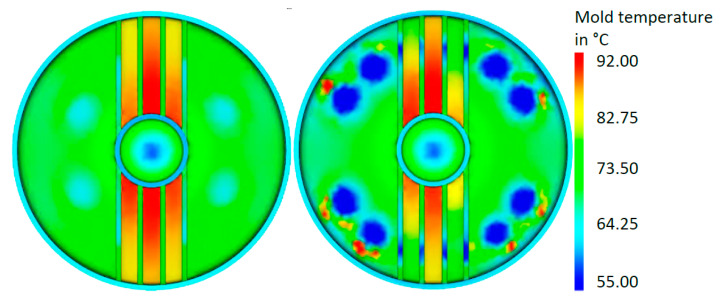
Temperature distribution on the lower surface of the cavity: initial configuration (**left**), optimized configuration (**right**).

A comparison of the temperature distribution in the initial configuration with that of the optimized solution is shown in Figure 12. Apparently, steep temperature gradients were encountered at the end points of the cooling channels while the high temperature values in the vicinity of the ribs improved only slightly. To sum up, the optimization of the cooling system of an injection molding tool, which retained a conventional cooling system, yielded only a rather insignificant optimization potential, so the findings of an optimized close-contour cooling system seem to be a worthwhile endeavor.

### 3.3. Optimized Close-Contour Cooling

**Figure 13:**
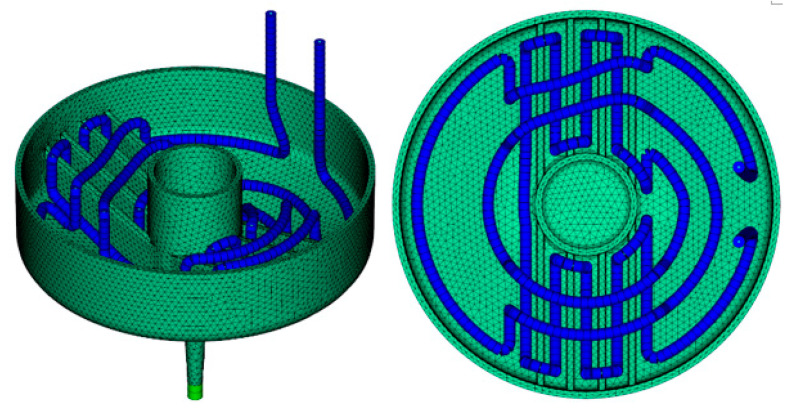
Mold part and final optimized configuration of the close-contour cooling system.

The optimal solution presented in the following section required 3 iterations with 28 simulations each (Figure 13). A comparison with the initial configuration (Figure 9) showed that the cooling channels outside the rib region had been shifted towards the inner and outer walls of the mold part. The start and end values of the optimization parameters are given in Table 7.

**Figure 14:**
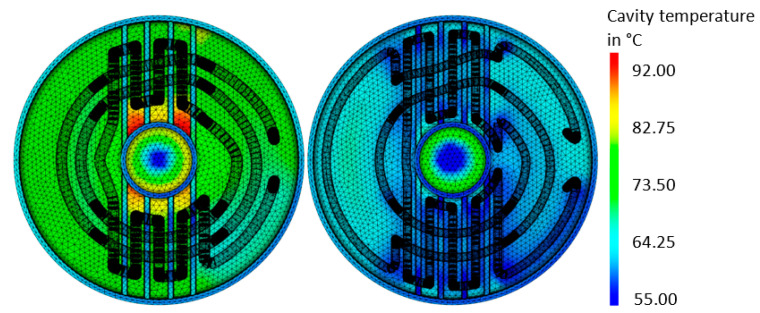
Temperature distribution in the tool cavity for close-contour cooling: a comparison between initial configuration (**left**) and optimized configuration (**right**).

**Figure 15:**
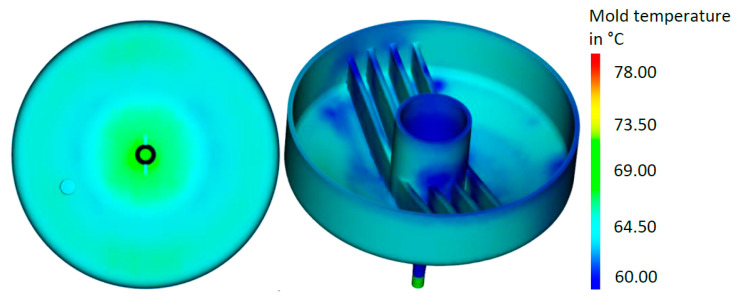
Temperature distribution on the surfaces of the mold part for optimized close-contour cooling.

**Figure 16:**
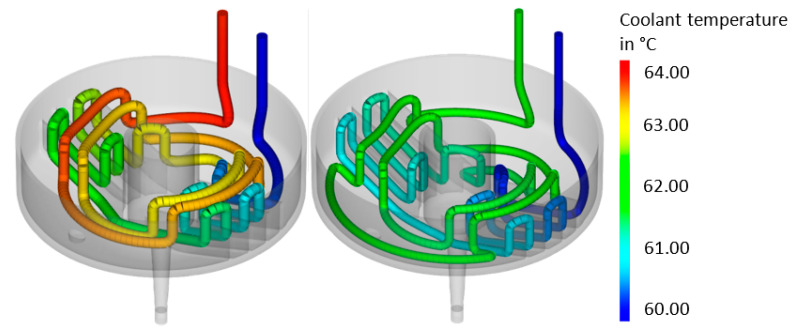
Temperature of the cooling medium for the initial (**left**) and optimized (**right**) close-contour cooling system.

The average temperature distribution in the tool for the close-contour cooling system is given in Figure 14. The results showed that an optimized cooling system can deliver significant potential in a reduced, more homogeneous temperature. The temperature distribution of the surfaces of the mold part is shown in Figure 15, the final optimized cooling channel distribution. Figure 16 includes the temperature of the cooling medium for the initial and optimized close-contour cooling system. It became obvious that by optimizing the cooling channel distribution, a significant reduction in the final outlet temperature was reached, which is beneficial for a homogeneous cooling rate in the molded part. This result was attributed to reduced channel diameters and the resultant higher flow rate.

### 3.4. Comparison of Results

**Figure 17:**
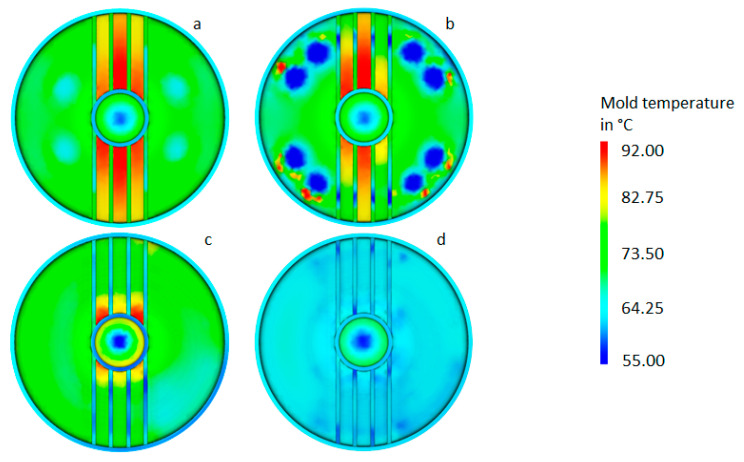
Graphical comparison of the temperature distribution on the lower tool surface: initial configuration (**a**) and optimized conventional configuration (**b**); initial close-contour configuration (**c**) and optimized close-contour cooling system (**d**).

For the investigated configurations, the simulation results are summarized in Table 8, and the corresponding temperature distribution on the lower tool surface is shown in Figure 17. Compared to the initial conventional configuration, the optimized conventional cooling system resulted in a slight reduction in the cycle time for a less homogeneous temperature distribution, which led leading to an increase in the local maximum temperature in the vicinity of the ribs, but this could not be considered a significant improvement. Conversely, the optimized close-contour design led to a reduced and more homogeneous temperature distribution. In addition, it produced a gain of about 15% in process cycle duration, including around 16 steps that are independent of a cooldown (see holding pressure and tool opening time of Table 1), that corresponded to a cooling time reduction of about 24%. This result correlated with the reduced channel diameters and the resultant higher flow rate. From a productivity point of view, the optimized close-contour design offered significant improvements compared to the initial configuration, even though the cycle time improved by only 4%. The overall better temperature distribution in the cavity obtained through the reduction of the peak temperature in the rib area hints at better mold part quality enabled by a more uniform shrinkage.

### 3.5. Economic Consideration

The reduced cycle time of a novel tooling design with optimized close-contour cooling directly translates into an increase in the production rate of an injection molding system. The economic impact was assessed using a very basic mathematical example wherein one year of non-stop production was assumed and the following fictitious values were used: mass of the molded part 70 g, material costs EUR 2.50 /kg, revenue per part EUR 0.50. The amount of manufactured parts was determined directly by dividing one year by the cycle time. Assuming that the fixed costs remained the same regardless of the productivity rate, the result is given in Table 9. Evidently the productivity rate increased significantly from an initial amount of 717,706 to 846,604 parts per year Consequently, the revenue increased by EUR 41,892, which shows both the quality and economic potential of optimized injection molding tools.

It is important to note that this basic mathematical example assumed the same lifetime for all the concepts, while in reality the conformal cooling mold would exhibit a shorter lifetime because reduced wall thicknesses over a non-negligible area at its top surface could lead to concentrations of stress. This comparative cost model does not account for variations in fixed costs such as energy consumption or storage capacity that come with a deep process change.

### 3.6. Proof of Concept Prototype Manufacturing

**Figure 18:**
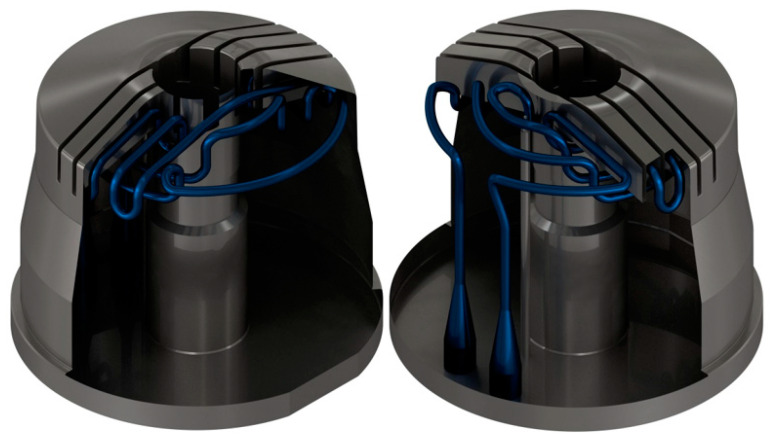
3D sectional model of the injection molding tool from two perspectives.

This section documents the additive manufacturing of a prototype of the mold core with optimized close-contour cooling channels as proof of concept. Manufacturing was performed on an EOS M290 machine (EOS GmbH, Krailling, Germany) equipped with a build volume of 250 × 250 × 325 mm and a 400 W Yb fiber laser, and the prototype was printed with AlSi10Mg. First, aluminum is commonly used for prototype manufacturing in injection molding, so a proof of concept manufactured with AlSi10Mg stands for the additive counterpart of a prototype made by conventional injection molding. Second, the additive manufacturing process is established at the Institute for Lightweight Construction and Design for this specific aluminum alloy. The 3D model was created using Autodesk Inventor (Autodesk Inc., Mill Valley, California, USA, v2019, 2018) and ANSA (BETA CAE Systems International AG, Root Switzerland, v19.1.1, 2019) software. Figure 18 provides an overview of the cooling channels together with a sectional view of the tool from two different perspectives. According to the literature, the channel diameters (Table 6) obtained by PBF without support structures yielded reasonable surface roughness for AlSi10Mg [27] that was used for the prototype and the tool steel [28].

**Figure 19:**
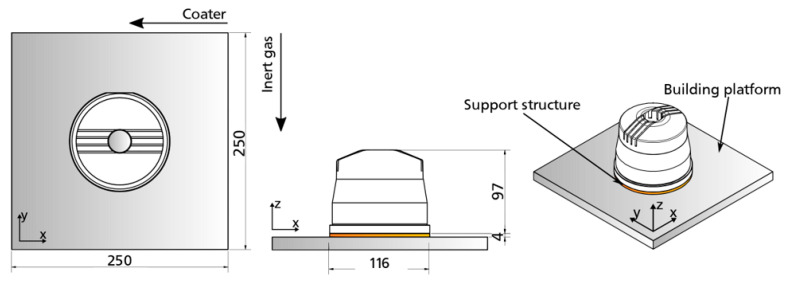
Sketch of the injection molding tool on the build platform of the PBF system.

**Figure 20:**
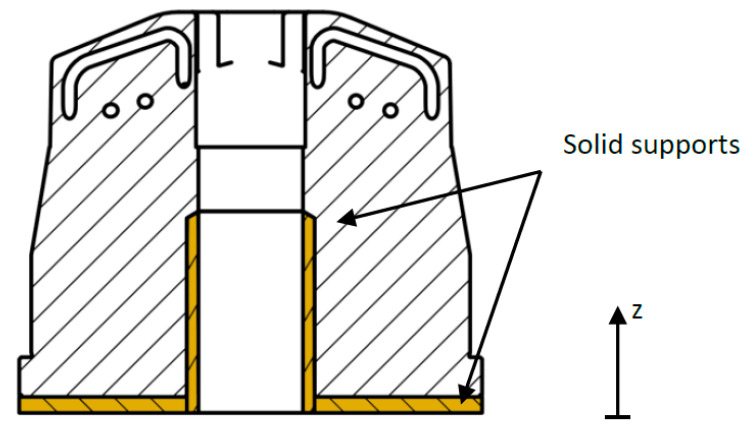
Support Structures.

**Figure 21:**
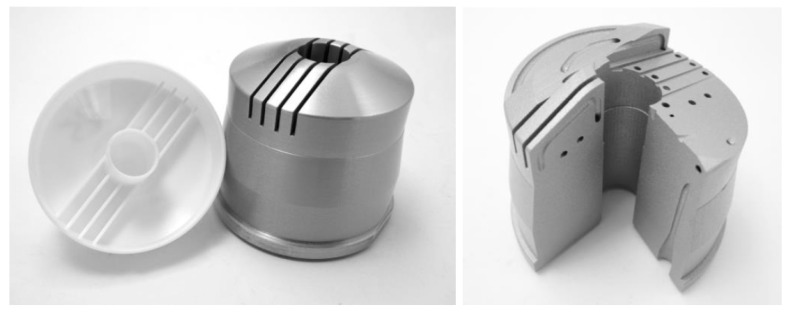
The final manufactured injection molding tool with the principle part (**left**) and sectional part exhibiting the integrated cooling channels (**right**).

Positioning of the part and supporting structures were performed by Materialise Magics (Materialize NV, Leuven, Belgium, v22.03, 2018) software, while parameter assignment required EOS Print (EOS GmbH, Krailling, Germany, v2.5.9, 2019) software. The part was manufactured upright in the center of the build platform, which enabled the lowest possible build height for time efficiency (34 h) and the minimum amount of support structures to involve as little rework as possible. To avoid geometrical inaccuracies induced by eigenstresses and inhomogeneous temperature gradients, solid supports based on extruded surfaces were used coupled with a build platform temperature of 180 °C. Moreover, all external surfaces were manufactured with an oversize of 0.5 mm to enable post-processing of all relevant surfaces. This consisted of turning all external surfaces with rotational symmetry, starting with the outer surface, and a precise CNC milling of the rib area by means of tool extension to achieve low external surface roughness. In the framework of this proof of concept, the inlets were not post-processed. Figure 19 depicts the part positioning on the building platform, while Figure 20 shows the support structures used. Figure 21 depicts the final manufactured injection molding tool with the principle molded part and a sectional part where the integrated cooling channels were clearly visible. The manufacturing showed no apparent irregularities, and the removal of excess powder from the cooling channels by suction proved to be unproblematic. Further characterization of the part was not covered in this paper.

## 4. Summary and Conclusions

This paper documented the numerical parameter optimization of a close-contour cooling system of an thermoplastic injection molding tool, based on numerical simulations, to optimize the overall cooling phase and thus reduce the cycle time and increase the part quality. An in-depth literature study revealed the driving parameters behind the performance of cooling channels as well as the main challenges when such optimized tools are manufactured using PBF. The investigations showed that optimizing cooling systems and transforming them from conventional designs to close-contour configurations contains significant potential for reducing cycle times, increasing productivity and improving part quality due to uniform temperature distributions and economic advantages. Lastly a prototype was manufactured, thus successfully enabling a proof of concept of the optimized tool.

Future investigations should be concerned with an optimized design taking into account post-cooling shrinkage. Furthermore, in-service tests on a steel tool should be performed to find the optimal porosity manufacturing parameters. Other interesting fields of research could be a comparison of close-contour cooling with other means of improving injection molding tools, such as implemented high conductivity inserts and, or the assessment of the potential and limits of additively manufactured tools compared to conventional ones and their impact on a cost-estimation model.

## Figures and Tables

**Table 1:** Process parameters for injection molding simulation.

Property	Value	Unit
Injection time	0.8	s
Volume and pressure control	99	%
Holding-pressure phase duration	10	s
Holding pressure	800	bar
Molten mass temperature	200	°C
Cooling medium temperature	60	°C
Cooling medium flow rate	10	L/min
Tool mold opening time	5	s

**Table 2:** Aims and boundary conditions for the performed simulations.

Condition	Value
Temperature difference in cooling agent	<3 °C
Minimum cycle time reduction	10%
Reduction of cavity temperature standard deviation	10%
Quasi-static loading	1000 bar

**Table 3:** Design variables for the optimization of the conventional cooling system.

Design Variable	Description	Start Value	Range	Unit
*n*_baffle_	Number of channels	8	4–12	-
*r*_base_	Radius of the base channel	40.8	30–45	mm
*l*_baffle_	Length of channels	80.0	50–80	mm
*ä*_baffle_	Channel angles	81.0	78–90	°

**Table 4:** Optimization parameters for the close-contour cooling system.

Parameter	Description	Initial Value	Range	Unit
*d*_inner_	Distance to inner hollow cylinder	10	3–15	mm
*d*_outer_	Distance to outer hollow cylinder	10	3–15	mm
*d_z_*	Distance to mold part in *z*-direction	5	3–15	mm
*d*_channel_	Diameter of cooling channel	4	3–5	mm
*r*_1_	Radius 1	25	20–30	mm
*r*_2_	Radius 2	35	25–40	mm

**Table 5:** Simulation results of the initial configuration.

Property		Value	Unit
Cycle Time		43.9	s
Cavity temperature	Mean value	70.0	°C
	Minimum value	55.9	°C
	Maximum value	92.1	°C
	Standard deviation	7.4	°C
Mold part temperature	Mean value	74.2	°C
	Maximum value	175.4	°C
	Standard deviation	12.7	°C

**Table 6:** Comparison of the simulation results of the initial configuration and the optimized solution.

Property		Initial	Optimized	Unit	Deviation
Cycle time		43.9	42.9	s	−2.3%
Cavity temperature	Mean value	70.0	68.7	°C	−1.9%
	Maximum value	92.1	97.6	°C	6.0%
	Standard deviation	7.4	7.4	°C	−0.0%

**Table 7:** Initial and final values for the optimization parameters for the close-contour cooling system.

Parameter	Description	Initial	Optimized	Unit
*d*_inner_	Distance to inner hollow cylinder	10	3.1	mm
*d*_outer_	Distance to outer hollow cylinder	10	4.5	mm
*d_z_*	Distance to mold part in *z*-direction	5	3.1	mm
*d*_channel_	Diameter of cooling channel	4	3.2	mm
*r*_1_	Radius 1	25	26.4	mm
*r*_2_	Radius 2	35	35.1	mm

**Table 8:** Comparison of the simulation results for the initial and optimized conventional configurations (**left**) with the initial and optimized close-contour configurations (**right**).

Configuration		Conventional Initial	Conventional Optimized	Close Contour Initial	Close Contour Optimized	Unit
Cycle time		43.9	42.9	38.9	37.2	s
Cavity temperature	Mean value	70.0	68.7	66.3	63.5	°C
	Maximum value	92.1	97.6	78.1	70.0	°C
	Standard deviation	7.4	7.4	2.9	1.7	°C

**Table 9:** Comparison of the productivity rate and the revenue for the initial standard configuration and the optimized close-contour cooling solution.

Cooling	Cycle Time	Parts per			Material Costs Per Year	Revenue Per Year
		Day	Month	Year		
Conventional	43.94 s	1966	58,990	717,706	125,599 €	233,254 €
Close-contour	37.25 s	2319	69,584	846,604	148,156 €	275,146 €
		Difference: +128,898			Difference: +41,892 €	

## Data Availability

Data sharing is not applicable for this article.
